# Yeast-Based High-Throughput Screens to Identify Novel Compounds Active against *Brugia malayi*

**DOI:** 10.1371/journal.pntd.0004401

**Published:** 2016-01-26

**Authors:** Elizabeth Bilsland, Daniel M. Bean, Eileen Devaney, Stephen G. Oliver

**Affiliations:** 1 Cambridge Systems Biology Centre and Department of Biochemistry, University of Cambridge, Cambridge, United Kingdom; 2 Department of Structural and Functional Biology, Institute of Biology, UNICAMP, Campinas, São Paulo, Brazil; 3 Institute of Biodiversity, Animal Health and Comparative Medicine, University of Glasgow, Glasgow, United Kingdom; McGill University, CANADA

## Abstract

**Background:**

Lymphatic filariasis is caused by the parasitic worms *Wuchereria bancrofti*, *Brugia malayi* or *B*. *timori*, which are transmitted via the bites from infected mosquitoes. Once in the human body, the parasites develop into adult worms in the lymphatic vessels, causing severe damage and swelling of the affected tissues. According to the World Health Organization, over 1.2 billion people in 58 countries are at risk of contracting lymphatic filariasis. Very few drugs are available to treat patients infected with these parasites, and these have low efficacy against the adult stages of the worms, which can live for 7–15 years in the human body. The requirement for annual treatment increases the risk of drug-resistant worms emerging, making it imperative to develop new drugs against these devastating diseases.

**Methodology/Principal Findings:**

We have developed a yeast-based, high-throughput screening system whereby essential yeast genes are replaced with their filarial or human counterparts. These strains are labeled with different fluorescent proteins to allow the simultaneous monitoring of strains with parasite or human genes in competition, and hence the identification of compounds that inhibit the parasite target without affecting its human ortholog. We constructed yeast strains expressing eight different *Brugia malayi* drug targets (as well as seven of their human counterparts), and performed medium-throughput drug screens for compounds that specifically inhibit the parasite enzymes. Using the Malaria Box collection (400 compounds), we identified nine filarial specific inhibitors and confirmed the antifilarial activity of five of these using *in vitro* assays against *Brugia pahangi*.

**Conclusions/Significance:**

We were able to functionally complement yeast deletions with eight different *Brugia malayi* enzymes that represent potential drug targets. We demonstrated that our yeast-based screening platform is efficient in identifying compounds that can discriminate between human and filarial enzymes. Hence, we are confident that we can extend our efforts to the construction of strains with further filarial targets (in particular for those species that cannot be cultivated in the laboratory), and perform high-throughput drug screens to identify specific inhibitors of the parasite enzymes. By establishing synergistic collaborations with researchers working directly on different parasitic worms, we aim to aid antihelmintic drug development for both human and veterinary infections.

## Author Summary

We have developed and validated a yeast-based high-throughput screening assay for the identification of specific inhibitors of filarial targets. We engineered yeast strains to functionally express parasite and human enzymes, labeling these with fluorescent proteins and growing them in competition in the presence of test compounds. These strains express different target proteins from *Brugia malayi* (as well as their human orthologs) and our results demonstrate that it is possible to identify compounds that can discriminate between filarial and human enzymes. Accordingly, we are confident that we can extend our assay to novel targets from *Brugia malayi* and other worms of medical and veterinary importance, and perform high-throughput screens to identify new drugs against different parasitic worms.

## Introduction

Lymphatic filariasis is a neglected tropical disease caused primarily by the parasitic nematodes *Wuchereria bancrofti* and *Brugia malayi*. The painful and disfiguring manifestations of this disease, also known as elephantiasis, can lead to permanent disability, causing an annual loss of approximately 5.5 million disability adjusted life years, affecting the poorest populations in Africa, Asia, and Latin America [1]. Current antifilarial therapies aim to eliminate filariasis through mass drug administration. However, in standard doses, the drugs used for this purpose (diethylcarbamazine, ivermectin and albendazole) are not effective against adult nematodes. As the adult worms can live in the human body for *ca*. 15 years [2], patients need to undergo multiple rounds of treatment, increasing not only the cost of therapy, but also the risk of drug-resistant worms emerging [3–5].

Filarial worms are difficult to cultivate *in vitro*, so adult worms for laboratory studies have to be obtained from animal models. Marcellino et al. [6] successfully developed a whole-plate, motion-based screen for monitoring drug activity against macroscopic parasites (WormAssay). This method was subsequently employed in screens against *B*. *malayi* [2], leading to the identification of the antifilarial activity of the FDA-approved drug auranofin. Unfortunately, there is no small animal model for other filarial worms, such as *W*. *bancrofti* [7] or *Onchocerca volvulus*; hence, there is a requirement for novel assays in the search for better treatments targeting filariasis cell-based assays also require extensive optimization). An alternative to parasite-based assays is to use *in vitro* drug screens based on protein targets. However, *in vitro* target-based assays require careful (and costly) optimization of the screening platform for each individual target protein to be tested, and provide no information on whether the drug is likely to be taken up by cells or whether it has general cytotoxicity.

To address these problems, we have developed and successfully validated a novel approach to high-throughput screens (HTS) for antiparasitic compounds using yeast [8,9]. Yeast cultures, which can be grown rapidly and at low cost, are ideal for use in automated screens [8–11]. Yeast cells are suitable hosts for the expression of nematode proteins [12–18], including enzymes essential for different life-cycle stages of the parasites, many of which cannot be propagated *in vitro* [17]. We engineered *Saccharomyces cerevisiae* strains to express either different parasite drug targets [9], or their equivalent human proteins, such that the growth of the yeast is dependent on the functioning of these heterologous proteins. We then transformed the engineered strains with plasmids expressing either CFP (cyan fluorescent protein), Venus (yellow fluorescent protein), Sapphire (blue fluorescent protein) or mCherry (red fluorescent protein), to enable their *in vivo* labeling.

Our engineered yeast strains are genetically identical, apart from expressing different heterologous drug targets and fluorescent labels that allow the growth of multiple strains to be followed in a single culture. These mixed cultures can be treated with chemical libraries to identify compounds capable of specifically inhibiting strains with the parasite targets but not their human counterparts. By these means, the drug sensitivity observed in a particular strain can be directly linked to the *in vivo* inhibition of the heterologous target protein. This approach has a number of significant advantages over conventional screens: it is very easy to set up for different drug targets; it is cheap, as the volumes used are very small and the yeast growth medium is inexpensive; it discriminates between compounds affecting parasite enzymes and human enzymes, and, by definition, active compounds must be able to enter living cells.

In this work, we evaluated the potential of such yeast-based drug screens in the identification of novel antifilarial compounds. We constructed yeast strains expressing different *B*. *malayi* target proteins, and used them to screen for novel inhibitors of filarial enzymes. We utilized a publicly available small-chemical library (400 Malarial Box compounds; http://www.mmv.org/malariabox) and identified compounds with significant inhibitory activity against the *B*. *malayi* enzymes, but little or no detectable activity against the equivalent human enzymes expressed in yeast. These first hit compounds were then validated *in vitro* against the closely related species, *Brugia pahangi* (continuously cultivated in our laboratory) with encouraging results, providing a proof of principle for this approach.

## Methods

### Ethics statement

All animal protocols were carried out in accordance with the guidelines of the UK Home Office, under the Animal (Scientific Procedures) Act 1986, following approval by the University of Glasgow Ethical Review Panel. Experiments were performed under the authority of the UK Home Office, project numbers 60/4448 and 60/3792.

### Plasmid constructs

The filarial enzymes selected for testing are listed in Table 1. The coding regions of Bm1_22900 (*Bm*NMT), Bm1_01925 (*Bm*PGK), Bm1_29130 (*Bm*TPI), Bm1_ 49000 (*Bm*PIS), Bm1_48165 (*Bm*SAH), Bm1_38705 (*Bm*SEC53), Bm1_11585 (*Bm*ADE13) and Bm1_23075 (*Bm*CDC8) were PCR-amplified from an adult *Brugia malayi* cDNA library, kindly donated by the Filariasis Research Reagent Resource Center (University of Georgia). These were cloned into the *Bam*HI-*Pst*I sites of pCM188 [19] to produce pCM*Bm*NMT, pCM*Bm*PGK, pCM*Bm*TPI, pCM*Bm*PIS, pCM*Bm*SAH, pCM*Bm*SEC53, pCM*Bm*ADE13 and pCM*Bm*CDC8. These constructs placed the heterologous genes under the control of the TetO2 promoter. Synthetic genes encoding *Brugia malayi* Bm1_33465 (*Bm*CDC21), Bm1_16500 (*Bm*KRS), Bm1_42945 (*Bm*MVD), Bm1_57600 (*Bm*RKI) and Bm1_16300 (*Bm*DYS) were synthesized by Geneart and sub-cloned into the *Bam*HI-*Pst*I sites of pCM188 [19] to produce pCM*Bm*CDC21, pCM*Bm*KRS, pCM*Bm*MVD, pCM*Bm*RK1, and pCM*Bm*DYS.

**Table 1:** Targets selected for yeast-based screens.

Target protein	*Brugia malayi*	*Wuchereria bancrofti*	*Homo sapiens*	Functions	Cognate yeast gene
N-myristoyltransferase (NMT)	Bm1_22900	WUBG_03313	O60551	Covalent attachment of myristic acid to the N-terminal glycine residue of several proteins, cellular growth and signal transduction	*NMT1*
phosphoglycerate kinase (PGK)	Bm1_01925	WUBG_03621	P00558	Glycolysis, energy metabolism	*PGK1*
triose-phosphate isomerase (TPI)	Bm1_29130	WUBG_07449	P60174	Glycolysis, energy metabolism	*TPI1*
adenosyl homocysteinase	Bm1_48165	WUBG_05842	P23526	Cellular lipid homoeostasis	*SAH1*
Inorganic pyrophosphatase	Bm1_16955	WUBG_14547	Q15181	Rapid exchange of oxygen from Pi with water	*IPP1*
Phospho mannomutase	Bm1_38705	WUBG_05311	O15305	Folding and glycosylation of secretory proteins	*SEC53*
thymidylate synthase	Bm1_33465	WUBG_07963	P04818	Biosynthesis of pyrimidine deoxyribonucleotides	*CDC21*
lysyl-tRNA synthetase	Bm1_16500	WUBG_09456	Q15046	Protein synthesis	*KRS1*
adenylosuccinate lyase	Bm1_11585	WUBG_03558	P30566	Purine nucleotide biosynthesis	*ADE13*
diphospho mevalonate decarboxylase	Bm1_42945	WUBG_07413	P53602	Biosynthesis of isoprenoids and sterols	*MVD1*
ribose-5-phosphate isomerase	Bm1_57600	WUBG_03052	P49247	Pentose phosphate pathway	*RKI1*
thymidylate kinase	Bm1_23075	WUBG_02750	P23919	Biosynthesis of pyrimidine deoxyribonucleotides	*CDC8*
deoxyhypusine synthase	Bm1_16300	WUBG_02450	P49366	Hypusine biosynthesis	*DYS1*
very-long-chain (3R)-3-hydroxyacyl-CoA dehydratase	Bm1_32340	WUBG_04779	Q6Y1H2	Sphingolipid biosynthesis and protein trafficking	*PHS1*
CDP-alcohol phosphatidyl transferase	Bm1_49000	WUBG_00951	O14735	Biosynthesis of phosphatidylinositol (glycolipid anchors for some of the plasma membrane proteins)	*PIS1*

The coding regions of human TPI1 (*Hs*TPI), CDIPT (*Hs*PIS), AHCYL1 (*Hs*SAHa), AHCY/SAHH (*Hs*SAHb), PMM2 (*Hs*SEC53), PUR8 (*Hs*ADE13), CDC8/DTYMK (*Hs*CDC8) and PPA1 (*Hs*IPP1a) were PCR amplified from a cerebellum cDNA library, kindly donated by Dr. Nianshu Zhang (University of Cambridge). These were cloned into the *Bam*HI-*Pst*I sites of pCM188 [19] to produce pCM*Hs*TPI, pCM*Hs*PIS, pCM*Hs*SAHa, pCM*Hs*SAHb, pCM*Hs*SEC53, pCM*Hs*ADE13, pCM*Hs*CDC8 and pCM*Hs*IPP1a. Synthetic genes encoding *Homo sapiens* TYMS (*Hs*CDC21), LysRS (*Hs*KRS), MVD (*Hs*MVD), and RPIA (*Hs*RKI) were synthesized by Geneart and sub-cloned into the *Bam*HI-*Pst*I sites of pCM188 [19] to produce pCM*Hs*CDC21, pCM*Hs*KRS, pCM*Hs*MVD, and pCM*Hs*RK1. Plasmid maps for new constructs developed in this work are available in Figs A to AF in S1 Text. Plasmids encoding human NMT2 and PGK1 (pCM*Hs*NMT and pCM*Hs*PGK) are described in Bilsland et al [9].

### Yeast strains

*PDR5* encodes the major drug export pump of *Saccharomyces cerevisiae*, hence we deleted *PDR5* in all of our yeast strains to increase their susceptibility to the test compounds. Deletion of the yeast *PDR5* coding sequence was performed as described previously [20].

pCM*Bm*NMT constructs were transformed into *nmt1Δ*::*Kan*MX*/NMT1 pdr5*Δ::*HisMX/PDR5* strains (BY4743 background [21]). pCM*BmPGK* constructs were transformed into *pgk1*Δ::*Kan*MX*/PGK1 pdr5*Δ::*His*MX*/PDR5* strains (BY4743 background). The same approach (transformation of the heterologous construct into a yeast strain heterozygous for a deletion mutant of the orthologous yeast gene and heterozygous for *pdr5*) was employed for all subsequent constructs. Heterozygous strains harbouring the heterologous constructs were sporulated and derived haploids were selected for growth assays and drug screens. Strain genotypes are described in Table A in S1 Text.

### Bioinformatics

*Saccharomyces cerevisiae*, *Brugia malayi* and *Homo sapiens* orthologues were selected based on data available at: http://inparanoid.sbc.su.se/cgi-bin/index.cgi. Multiple protein sequence alignments were performed using https://www.ebi.ac.uk/Tools/msa/, creating a pairwise identity matrix between each protein orthologue.

### Growth assays

Yeast strains were grown in 2.5 mL YPD (1% yeast extract, 2% peptone, 2% glucose) cultures overnight at 30°C. Cultures were diluted 100 times in fresh YPD with 0, 5 or 10 mg/L doxycycline to tune-down the expression of the target protein from the TetO2 promoter. Clear 384-well plates (Corning) were then filled with 70 μL of each diluted culture (in triplicate). Plates were incubated in a BMG Optima plate reader and OD_595_ measures for each culture were acquired every 15 minutes. Growth scores were obtained by calculating the maximum exponential growth rate for each culture, and then multiplying this value by the yield of the culture (yield = maximum OD_595_—minimum OD_595_). Growth scores of the yeast strains dependent for growth on either the *B*. *malayi* or human coding sequence for the target enzyme were divided by the score for the corresponding wild-type strains (BY4741 or BY4742), to estimate their relative growth.

### Drug screens

Yeast strains were transformed with fluorescent plasmids expressing one of the following fluorescent proteins mCherry (yEp_Cherry_LEU2), Venus (yEp_venus_LEU2), Sapphire (yEp_sapphire_LEU2) or CFP (yEp_CFP_LEU2) [8]. This allowed us to monitor, in real time, the growth of 2–4 different yeast strains growing in competition. Fluorescently labelled strains were grown in 2.5 mL YNB (1, 7 g/l yeast nitrogen base, 5 g/L ammonium sulphate, 2% glucose, and amino acid supplements) overnight at 30°C. Cultures were pooled and diluted 50 times in fresh YNB. An aliquot (35 μL) of the diluted pooled-culture was added to each well of black 384-well plates, together with 35 μL of YNB with 20 μM of each test compound (final 70 μL of 1:100 cultures with 10 μM of the test compound). Each experiment was run in quadruplicate, with two replicates using one combination of fluorescent markers, and two replicates using the alternate combination, in order to minimize false-positive results. Plates were incubated in a BMG Optima plate reader and fluorescence measured every 25 minutes, for 48 hours, in 4 different channels: Venus (excitation 500 nm/emission 540 nm), CFP (440 nm/490 nm), Sapphire (405 nm/510 nm) and mCherry (580 nm/612 nm). We confirmed each of our hits by generating dose:response curves with 0, 2, 10 and 50 μM of each hit compound.

### Curve fitting

Time-series fluorescence data from each channel was analyzed using a fitting procedure written in R, based on the model-free spline method of Kahm *et al*. [22]. The first 26 fluorescence measurements were used for analysis (corresponding to 26.66 hours of incubation); beyond this time, fluorescence decayed due to photo-bleaching. Specifically, a smoothed spline was fitted to the raw data for each channel and the first derivative through each point of the fit calculated. The highest derivative was taken as the maximum exponential growth rate (μ). This gradient was extrapolated back towards the start of the growth curve by fitting a straight line of the form y = mx + c. The lag phase duration (λ) was estimated by finding the intercept of this line and the baseline fluorescence from the start of the experiment. The fluorescence yield was calculated by subtracting the baseline fluorescence from the maximum value of the fit. These parameters are summarized in Fig AG in S1 Text.

### Normalization and scoring

Each compound was scored according to:

$$Score\;=\;\frac{rate\;\times \;yield}{lag}$$

The average score for each compound was normalized to the average score for the DMSO control from the corresponding row in the plate. Compounds were scored for each pool (marker swap) independently. A compound was considered a hit when the relative growth score, in both marker combinations, of the recombinant strains expressing the parasite target protein was significantly lower than the growth score of the strain expressing the human enzyme.

### Validation in *Brugia pahangi*

Female worms of *B*. *pahangi* were recovered from infected jirds (the rodent, *Meriones unguiculatus*) approximately four months post-infection, exactly as described previously [23]. Worms were washed once in Hank’s Balanced Salt Solution and then cultured individually in 24-well tissue culture plates containing 2.0 ml of RMPI-1640 (Invitrogen, Cat No: 52400) supplemented with 5% heat-inactivated fetal bovine serum, 1% glucose and 100 units per mL of penicillin/streptomycin (all Invitrogen) for 24 hours to monitor viability and microfilarial output. Healthy worms were then selected for drug testing. Initially, each compound was tested in triplicate at concentrations of 50 μM and 10 μM. Control wells contained the appropriate concentration of the DMSO solvent. Parasites were also exposed to geldanamycin, a known Hsp90 inhibitor, at the same concentrations, as a positive control. All cultures were maintained at 37°C in 5% v/v CO_2_ in air and were monitored daily for viability. In a second experiment, adult worms were exposed to a wider concentration range of three selected drugs that had proved active in the first experiment. These were MMV396794, MMV665941 and MMV666022. Each drug was tested in triplicate at 25, 10, 5 and 2.5 μM.

## Results

### Target selection

We initially selected 15 enzymes (Table 1) to test as anti-filarial targets according to the following criteria: highly expressed in the adult stages of the parasite; likely to be essential for the viability of the nematode in the human host; and have an essential yeast ortholog. As drugs such as DEC and ivermectin have potent microfilaricidal activity, we chose to focus on adult parasites to control the stages not affected by current therapies. Furthermore, adult worms are the cause of much of the pathology associated with lymphatic filariasis [24].

### Cloning of the *Brugia malayi* targets and human counterparts

We obtained *Wuchereria bancrofti* and *Brugia malayi* cDNA libraries, prepared from mRNA extracted from adult stages of the nematodes, from the Filariasis Research Reagent Resource Center (The University of Georgia). These cDNA libraries were used as templates for amplification by polymerase chain reaction (PCR), and cloning into yeast plasmids.

From the outset of our work, we noticed that the publicly available genome sequence of *W*. *bancrofti* contains multiple gaps, which frequently overlapped with our target genes. This made the design of primers for coding sequence (cds) amplification problematic. On the other hand, the genome sequence of *B*. *malayi* is very well annotated, facilitating the cloning procedure. Hence, we focused most of our cloning efforts on *B*. *malayi* targets. In spite of this, after multiple attempts and primer designs, we failed to amplify seven of the intended *B*. *malayi* targets (Table 2). This was probably due to either the absence of that particular cDNA in the libraries or to errors in the annotated sequence. The problem due to the low coverage of our cDNA library was overcome by synthesizing DNA encoding each of the selected *Brugia* targets to allow their expression in yeast. We identified two instances of errors in the published *Brugia* genome sequence by either sequencing our clones derived from cDNA (*Bm*SAH), or by phenotyping strains expressing a synthetic cds based on the predicted sequence of the *Bm*MVD gene product.

**Table 2:** *Brugia malayi* drug targets. *B*. *malayi* targets selected for our screening pipeline (column 1); yeast and human orthologs (columns 2 and 3, respectively); outcome of the cloning efforts of *B*. *malayi* (*Bm*) or human (*Hs*) genes (columns 4 and 5); outcome of the functional complementation in yeast (columns 6 and 7), where % complementation = 100 * growth score of strains with the heterologous target/ growth score of wild-type strains; source of coding region for each clone (column 8).

Brugia *malayi*	Yeast Gene	HumanGene	*Bm* cloning	*Hs* cloning	*Bm* compl (%)	*Hs* compl (%)	OBS
Bm1_22900	*NMT1*	NMT2	Yes	Yes	Yes (96)	Yes (100)	cDNA
Bm1_01925	*PGK1*	PGK1	Yes	Yes	Yes (139.)	Yes (110)	cDNA
Bm1_29130	*TPI1*	TPI1	Yes	Yes	Yes (84)	Yes (77)	cDNA
Bm1_49000	*PIS1*	CDIPT	Yes	Yes	Yes (86)	Yes (98)	cDNA
Bm1_48165	*SAH1*	AHCYL1 (A)	Yes	Yes	Yes (83)	Yes (103.)	cDNA
Bm1_48165	*SAH1*	AHCY, SAHH (B)	-	Yes	-	Yes (88)	cDNA
Bm1_48165	*SAH1*	AHCYL2 (C)	-	No	-		cDNA
Bm1_38705	*SEC53*	PMM2	Yes	Yes	No (0)	No (0)	cDNA
Bm1_11585	*ADE13*	ADSL	Yes	Yes	No (0)	No (0)	cDNA
Bm1_23075	*CDC8*	CDC8, DTYMK	Yes	Yes	No (0)	Yes (72)	cDNA
Bm1_16955	*IPP1*	PPA1 (A)	No	Yes		Yes (82)	cDNA
Bm1_16955	*IPP1*	PPA2 (B)	-	Yes	-	No (0)	cDNA
Bm1_16955	*IPP1*	LHPP (C)	-	Yes	-	No (0)	cDNA
Bm1_33465	*CDC21*	TYMS	Yes	Yes	Yes (94)	Yes (69)	Synthetic
Bm1_16500	*KRS1*	KARS	Yes	Yes	No (0)	No (0)	Synthetic
Bm1_42945	*MVD1*	MVD1	Yes	Yes	No (0)	Yes (88)	Synthetic
Bm1_57600	*RKI1*	RPIA	Yes	Yes	Yes (78)	Yes (117.)	Synthetic
Bm1_16300	*DYS1*	DHPS	Yes	No	Yes (91)	-	Synthetic
Bm1_32340	*PHS1*	PTPLa	No	Yes	-	No (0)	
Bm1_32340	*PHS1*	PTPLb	-	No	-	-	

**Fig 1:**
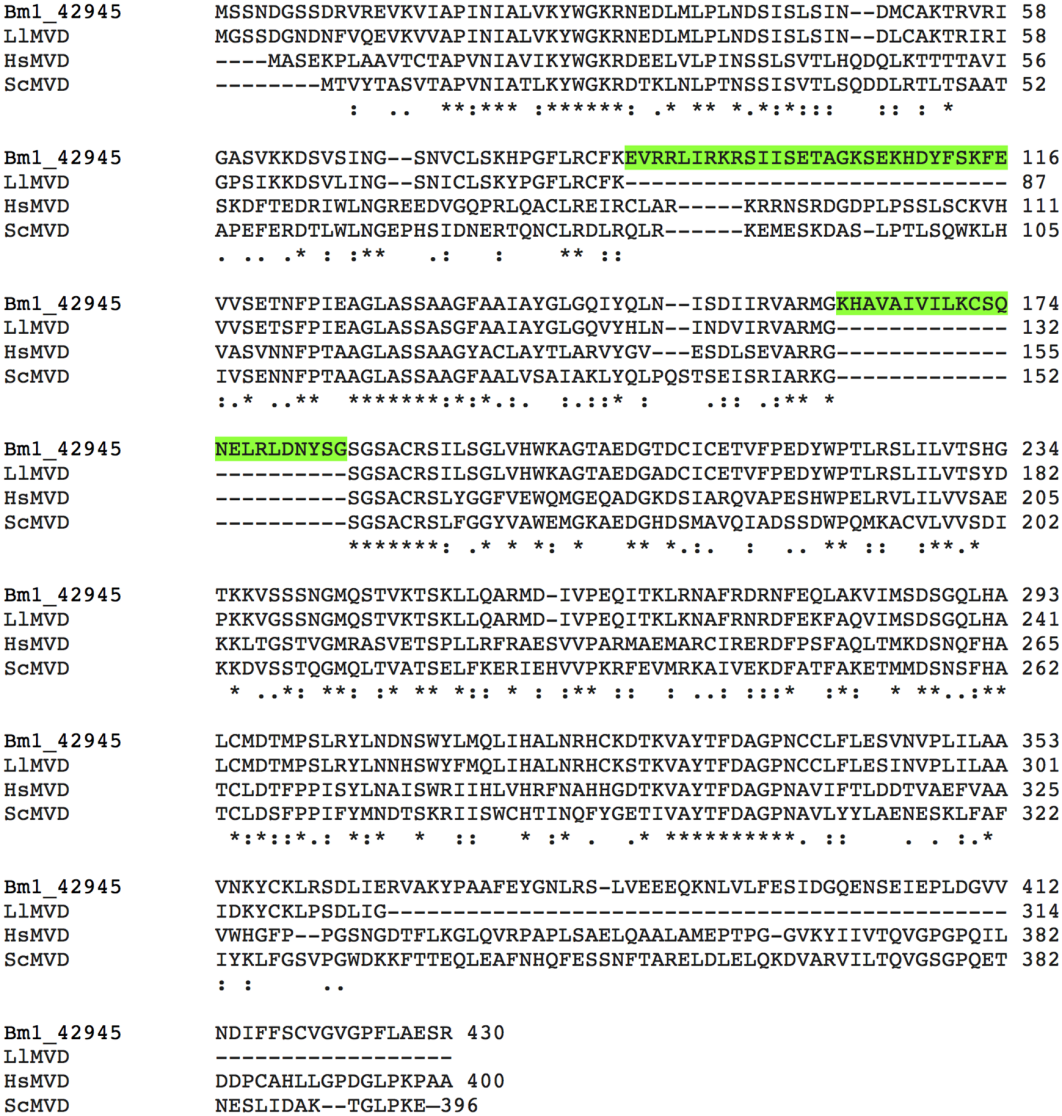
CLUSTAL 2.1 multiple sequence alignment of diphospho mevalonate decarboxylases from *Brugia malayi* (*Bm*MVD), *Loa loa* (*Ll*MVD), *Homo sapiens* (*Hs*MVD) and *Saccharomyces cerevisiae* (*Sc*MVD), highlighting two regions in the published *Bm*MVD sequences that diverge from the consensus between the orthologous protein sequences. These could be due to the presence of different splice variants of the *Brugia* enzyme, natural insertions or errors in the published sequence.* conserved residues;: chemically conserved changes;. non-conserved changes.

We observed that the synthetic human MVD could complement the yeast deletion very successfully (88% of wild-type growth), whereas the synthetic *B*.*malayi* MVD could not. Alignment of the publicly available *B*. *malayi*, *Loa loa* (eye worm), human, and yeast sequences, demonstrated that the *B*. *malayi* sequence diverged from those of the other three species, strongly suggesting either a non-conserved insertion in the *Brugia malayi* protein (which leads to the loss of function of the heterologous protein in yeast) or a problem with genome assembly of the published *B*. *malayi* sequence (Fig 1).

**Fig 2:**
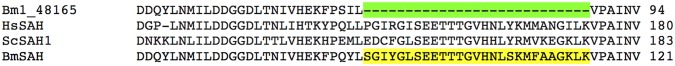
Partial CLUSTAL 2.1 multiple sequence alignment of publicly available *Brugia malayi* (Bm1_48165), *Homo sapiens* (*Hs*SAH), *Saccharomyces cerevisiae* (*Sc*SAH), and our cloned *Brugia malayi* (*Bm*SAH) S-adenosyl homocysteinases, showing the “insert” missing from the publicly available nematode protein sequence. The absence of the 27 amino acids in the publicly available *Brugia* sequence (Bm1_48165) could indicate a splice variant of the enzyme or simply a problem with the genome assembly.

Similarly, sequencing of the cloned *B*. *malayi* adenosylhomocysteinase (Bm1_48165, *Bm*SAH), demonstrated an insertion of 27 amino-acids (aa) between aa 88 and 89 of Bm1_48165. Eight independent *Bm*SAH plasmids were constructed and sequenced and the same insertion was always detected. As the same insertion is present in both yeast and human SAH (Fig 2), it suggests that there may be an error in the *B*. *malayi* genome sequence, or that *B*. *malayi* encodes more than one splice variant of the enzyme. Furthermore, the encoded protein from our clone is enzymatically functional in yeast, complementing the deletion of the orthologous yeast gene.

**Fig 3:**
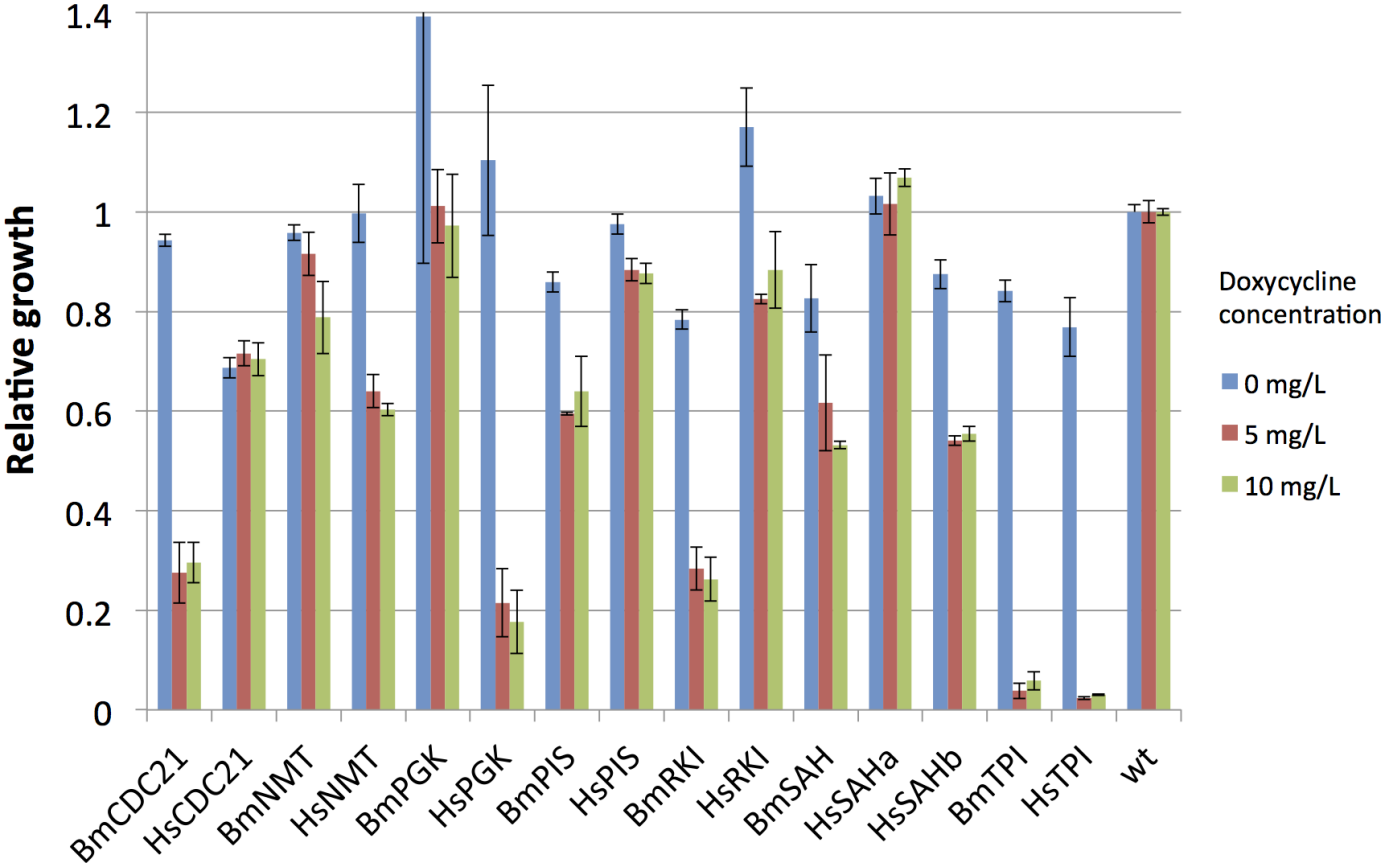
Functional complementation of yeast deletions by *Brugia malayi* (Bm) or *Homo sapiens* (Hs) orthologous genes. Relative growth of yeast strains expressing the parasite or human target compared to the growth of yeast strains expressing the native gene. Blue bars indicate the full expression of the heterologous genes from the TetO2 promoter, and red and green bars indicate the growth of strains with a reduced expression of the essential enzyme.

We constructed seven additional strains where *B*. *malayi* gene products (Bm1_22900/*Bm*NMT, Bm1_01925/*Bm*PGK, Bm1_29130/*Bm*TPI, Bm1_49000/*Bm*PIS, Bm1_33465/*Bm*CDC21, Bm1_57600/*Bm*RKI, Bm1_16300/*Bm*DYS) were able to complement the essential functions of the yeast orthologous gene. The heterologous genes were cloned into the yeast expression vectors under the regulation of the TetO2 promoter [25]. This promoter is constitutively on; however, by adding doxycycline to the growth medium, it is possible to tune-down the expression of the construct. Decreasing the expression of the target enzyme facilitates the growth inhibition by the test compounds (less target = more efficient inhibition). We have previously tested the inhibition of expression from the TetO2 promoter by addition of 1, 2, 5, 10, 20, 50 or 100 mg/L of doxycycline [9] and found that 5 and 10 mg/L would be ideal for the current work. Hence, we performed growth assays with each of the heterologous strains in medium with 0, 5 or 10 mg/L of antibiotic (Fig 3). However, we noticed that most of our strains require the full expression of the heterologous targets for optimum growth. Hence, drug screens employing the strains expressing *B*. *malayi* targets were performed in the absence of doxycycline.

**Fig 4:**
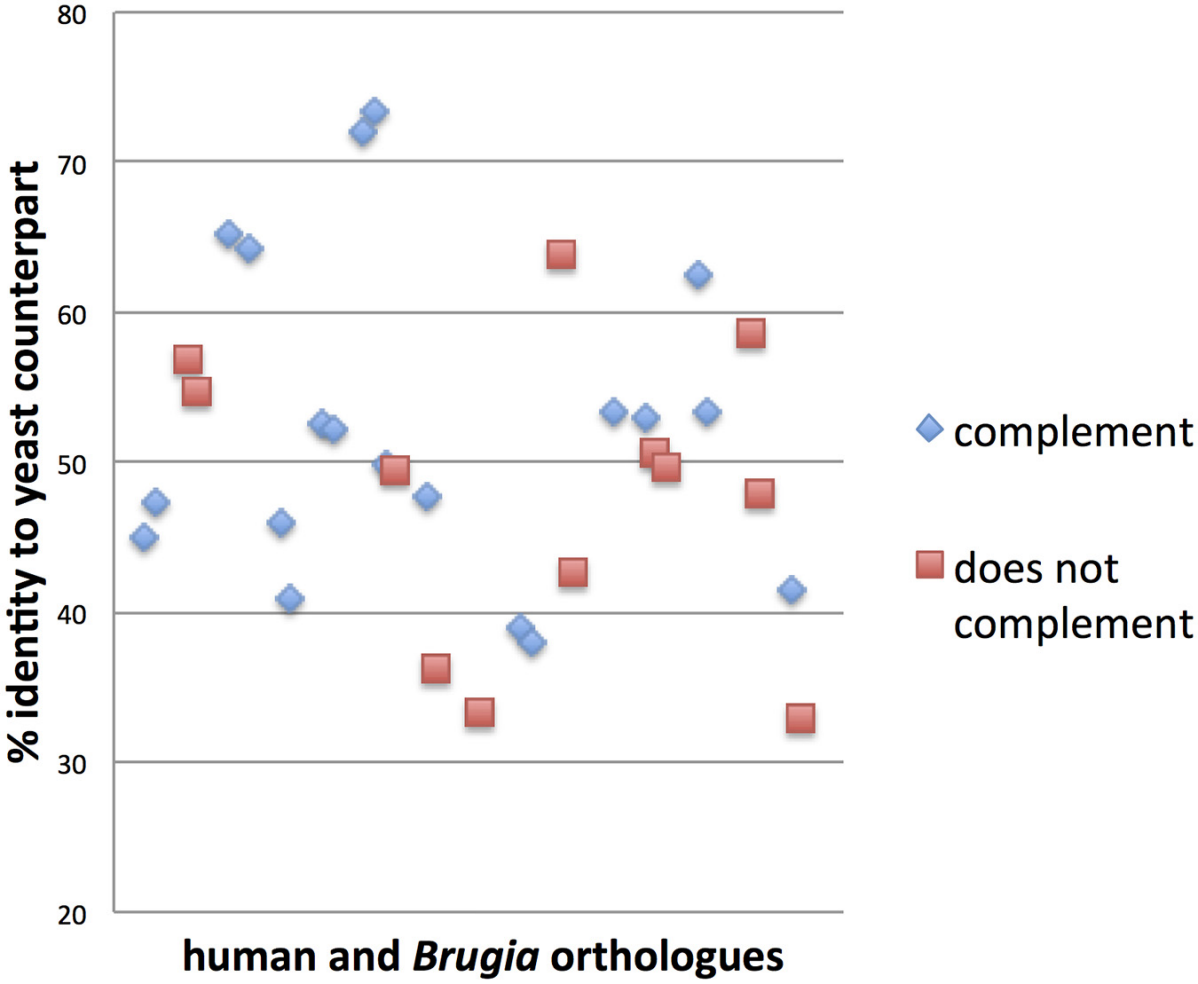
Scatter plot showing no correlation between the percentage identity between the human (*Homo sapiens*) or *Brugia* malayi proteins that do or do not complement the essential functions of the yeast (*Saccharomyces cerevisiae*) orthologues. The average protein identity (in %) between heterologous proteins able to complement the yeast deletions was 52.5 ± 10.6, whereas the identity between heterologous proteins NOT able to complement the yeast deletions was 48.1 ± 10.0.

In addition to *Bm*MVD, four other *B*. *malayi* clones constructed in our studies failed to complement the essential functions of their cognate yeast genes; these were: Bm1_38705 (*Bm*SEC53), Bm1_16500 (*Bm*KRS1), Bm1_11585 (*Bm*ADE13) and Bm1_23075 (*Bm*CDC8). With a view to establishing a sequence-similarity cut-off on which to base the selection of new targets, we calculated the percentage identity between yeast and filarial proteins that could successfully replace each other and those that did not. We found no clear correlation between sequence similarity and functional complementation; therefore, with the data collected so far, we cannot predict which filarial enzymes will be functional in yeast (Fig 4).

### Yeast screens established for five *Brugia* target proteins

The screening method developed in our laboratory allows the simultaneous screening of up to three parasite targets and of the human counterpart in a single assay [8,9]. Hence, to make best use of the screening efforts, we included yeast strains expressing *Schistosoma mansoni* PGK [9], *S*. *mansoni* NMT [9], *Homo sapiens* NMT [9], *H*. *sapiens* PGK [9], *H*. *sapiens* TPI, *H*. *sapiens* PIS and *H*. *sapiens* SAHa and SAHb (S1 Text). These eight strains, as well as the five strains expressing the corresponding *Brugia malayi* targets (Table 2), were labeled by expression of fluorescent proteins and screened against each of the 400 compounds from the Malaria Box collection at a concentration of 10 μM. Comparing the growth of yeast strains expressing either parasite or human enzymes, we identified a number of compounds that specifically inhibited the growth of strains dependent on the parasite enzymes (Table 3). We then performed dose:response experiments with 0, 2, 10 or 50 μM of each hit compound to confirm our NMT, PGK and PIS hits, and confirmed the specificity of the compounds at the indicated concentrations (Table 3, last column).

**Table 3:** List of compounds that specifically inhibit the parasite targets at 10 μM: Bm = *Brugia malayi*, Sm = *Schistosoma mansoni*, NMT = N-myristoyl transferase, PGK = phosphoglycerate kinase, PIS = inositol 3-phosphatidyltransferase, SAH = S-adenosylhomocysteinase. Smiles, or simplified molecular-input line-entry system, is a line notation for describing the structure of different chemical species. Ratio indicates the specificity of the compound for the parasite target = growth score for yeast strains expressing the parasite enzyme/growth score for the yeast strains expressing the human counterpart, Hence, a ratio of 1 indicates the absence of discrimination between parasite and human target, a ratio >1 indicates that the human enzyme is inhibited more than the parasite enzyme, and a ratio <1 indicates a specific inhibition of the parasite target. The last column indicates the concentration (in μM) in which the compounds specifically inhibited one or both of the parasite targets in dose-response experiments.

Malaria Box compound	Smiles	Target	Ratio at 10 uM	Inhibition confirmed at [] uM
MMV665941	CN(C)c1ccc(cc1)C(O)(c2ccc(cc2)N(C)C)c3ccc(cc3)N(C)C	*Bm*NMT/*Sm*NMT	0.17	10, 50
MMV009127	CC1CCN(Cc2c(O)ccc3C = C(C (= O)Oc23)c4nc5ccccc5s4)CC1	*Bm*PGK/*Sm*PGK	0.47	2, 10, 50
MMV666597	CCCCCCc1cc2C = C(C (= Nc3ccccc3C)Oc2cc1O)c4nc5ccccc5[nH]4	*Bm*PGK/*Sm*PGK	0.58	10, 50
MMV396797	n1(nc(C)c2c3ccc(OC)c(OC)c3)c2ncc(C#N)c1N	*Bm*PIS	0.74	50
MMV011259	n1(n2)c(nc(C)cc1Nc(cc(C)cc3C)c3)nc2C(F)(F)F	*Bm*SAH	0.51	-
MMV665843	Oc1c2CCCCc2nc3ccccc13	*Bm*SAH	0.84	-
MMV666022	COc1ccc(cc1)C (= O)NC(c2ccccc2Cl)c3cc(Cl)c4cccnc4c3O	*Bm*SAH	0.77	-
MMV006706	n1(c2c(cccc2)n3)c3c(C#N)c(C)cc1N(CC4)CCN4C5CCCC5	*Bm*SAH	0.85	-
MMV396794	c1(Cl)c(ccc(Cl)c1)OCC(O)CNC(C)(C)CC(C)(C)C	*Bm*SAH	0.88	

### Validation of *Brugia malayi* hits in *Brugia pahangi*

**Fig 5:**
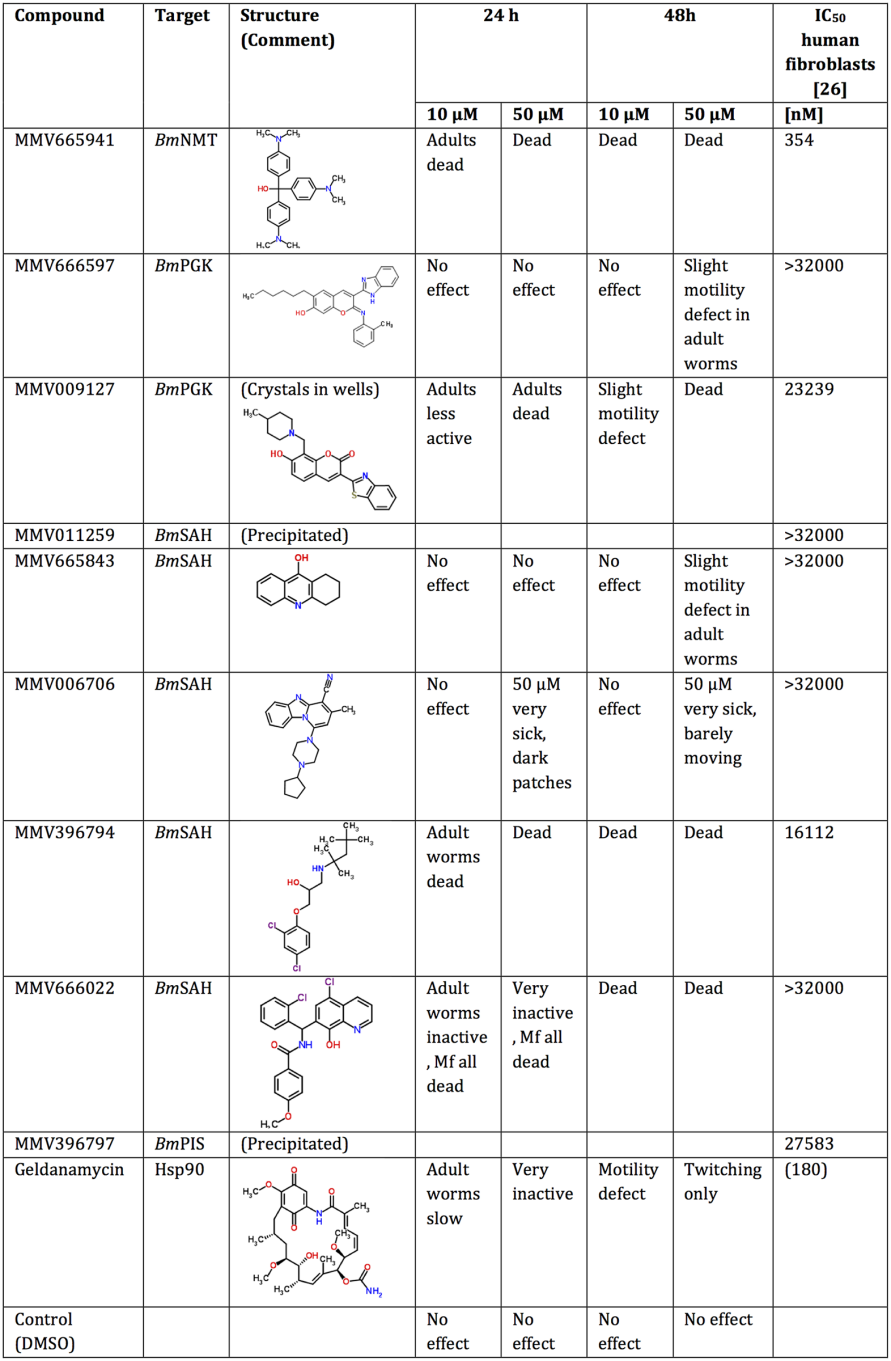
Validation of the hit compounds using *Brugia pahangi* adult female worms. Chemical structures were obtained from www.chemspider.com. The IC_50_ for each of the compounds in human fibroblasts (MRC5) is shown in the last column [26]. The IC_50_ of Geldanamycin against different human cell lines is a consensus of multiple data available in the literature (www.medchemexpress.com/Geldanamycin.html).

We performed drug-sensitivity assays of our nine *Brugia malayi* hits using adult *Brugia pahangi* worms. Two of the compounds were not soluble in the test conditions, but four of the soluble compounds killed the worms at concentrations of 10 or 50 μM. Furthermore, the three remaining compounds compromised the motility of *B*. *pahangi* (Fig 5). Of the four compounds that had pronounced effects on adult worms at 24h incubation, two had immediate effects on adult viability. For MMV396794 and MMV665941, adult worms were dead within 3h of incubation at 50 μM concentration. These two compounds and MMV666022 were re-tested over a wider range of concentrations starting at 25 μM. The results were very similar, with worms incubated in MMV MMV396794 and MMV665941 at 25 μM dying within 3h of exposure. At 10 μM, all worms were dead in MMV665941 by 6h of exposure and at 5.0 and 2.5 μM were tightly coiled, although still motile at 6h, but dead by 24h. Compound MMV396794 killed all worms at 10 μM by 24h, with worms at lower concentrations being sluggish at 24h, although still alive. MMV666022 was the least active compound—although, after 48h, worms were dying at 25 and 10 μM, they were largely unaffected at 5 or 2.5 μM. Considering that the compounds used in our screens (Malaria Box) have low toxicity to human cells, this initial validation was extremely encouraging.

## Discussion

We previously developed and validated an efficient high-throughput screening method for the identification of compounds that selectively inhibit the activity of parasite proteins that are candidate drug targets, but have little effect on the orthologous human proteins [8,9,11]. Most of our initial screens were performed with targets from protozoan parasites (*Plasmodium*, *Trypanosoma*, *Leishmania*) that are evolutionarily very distant from the human host. In the present work, we evaluated the suitability of our approach for identifying inhibitors of enzymes from a metazoan animal, the nematode *B*. *malayi*.

Our assay is based on replacing essential yeast genes with their parasite or human counterparts, to allow the identification of compounds that inhibit the growth of yeast cells expressing the parasite target but not the growth of yeast strains expressing their human counterpart. This discrimination is very efficient for targets from parasites that are only distantly related to humans. Therefore, at the outset of our studies, we were unsure as to whether we would identify any compound capable of inhibiting metazoan targets such as *Brugia*, without also inhibiting the human ortholog.

Through the construction of yeast strains expressing the heterologous drug targets, we identified two instances where the publicly available *B*. *malayi* protein sequences are incorrect (Bm1_48165 and Bm1_42945), and have made a small contribution towards improving the annotation of the parasite genome. We also created tools for further studies of the functions of eight different *Brugia* enzymes.

It was gratifying to note that even working with a small library of compounds (400 Malaria Box drugs), it was possible to find compounds that could discriminate between human and metazoan pathogen enzymes. Most importantly, the compounds identified in our yeast-based screens can kill *Brugia* parasites *in vitro*, or inhibit their motility. Further studies will be necessary to follow up on these findings and titrate minimal effective concentrations of drug with activity against adult filarial worms. Interestingly, inhibition of filarial NMT proved to be very effective in our screen, confirming previous data of Galvin et al [27]. In that study, NMT was shown to be essential for viability in both *B*. *malayi* and in the free-living model nematode *Caenorhabditis elegans*. Hence, we are confident of the potential of our approach in pre-selecting novel compounds against metazoan parasites, and will be able to extend our screens to targets from other parasitic nematode species. Recent studies on the systematic humanization of yeast strains [28] will be particularly helpfully in suggesting proteins that can successfully complement essential yeast deletions.

In conclusion, our yeast-based screen offers many advantages over organism-based screens as a first pass for identifying compounds with activity against key filarial targets. Future studies will screen for inhibitors of additional essential targets, and extend the screens to larger chemical libraries in the search for much-needed novel nematicides.

### Accession numbers/ID numbers for *Brugia malayi* targets used in this study

Bm1_22900, Bm1_01925, Bm1_29130, Bm1_48165, Bm1_16955, Bm1_38705, Bm1_33465, Bm1_16500, Bm1_11585, Bm1_42945, Bm1_57600, Bm1_23075, Bm1_16300, Bm1_32340, Bm1_49000.

## Supporting Information

S1 Text Supplementary information file. Synthetic gene sequences, plasmid maps **(**Fig A to Fig AF) and genotypes (Table A) of strains constructed or described in this work. Parameters extracted from fluorescent data (Fig AG), where the black line represents the fitted spline curve, the fluorescence yield is the difference between the baseline (dashed red line) and maximum (dashed green line), the lag duration, λ, is the intercept of the baseline and the maximum exponential growth rate, μ (dashed blue line).
(DOCX)
