# Supplementary material for: Yeast-Based High-Throughput Screens to Identify Novel Compounds Active against Brugia malayi
Source: PLoS Negl Trop Dis. 2016 Jan 26;10(1):e0004401. doi: 10.1371/journal.pntd.0004401 (PMC4727890; doi:10.1371/journal.pntd.0004401)
Supplement: S1 Text — Synthetic gene sequences, plasmid maps (Fig A to Fig AF) and genotypes (Table A) of strains constructed or described in this work. Parameters extracted from fluorescent data (Fig AG), where the black line represents the fitted spline curve, the fluorescence yield is the difference between the baseline (dashed red line) and maximum (dashed green line), the lag duration, λ, is the intercept of the baseline and the maximum exponential growth rate, μ (dashed blue line). (DOCX) [file pntd.0004401.s001.docx]

Yeast-based high-throughput screens to identify novel compounds active against *Brugia malayi*

Elizabeth Bilsland^1,2^, Daniel M. Bean^1^, Eileen Devaney^3^, Stephen G. Oliver^1^

1. Department of Biochemistry, University of Cambridge, Cambridge, UK

2. Department of Structural and Functional Biology, Institute of Biology, UNICAMP, Campinas –SP, Brazil

3. Institute of Biodiversity, Animal Health and Comparative Medicine, University of Glasgow, Glasgow, UK

# 1. Synthetic Genes with codon usage optimized for expression in *Saccharomyces cerevisiae*

Synthetic coding region of each gene flanked by *BamHI* and *PstI* restriction sites (highlighted in yellow).

## Synthetic *HsMVD*

GGATCCATGGCTTCTGAAAAACCATTG

GCTGCTGTTACTTGTACTGCTCCAGTTAATATTGCCGTTATTAAGTACTGGGGTAAGAGAGATGAAGAATTGGTTTTGC

CAATCAACTCCTCTTTGTCTGTTACCTTGCATCAAGACCAATTGAAAACTACTACCACCGCCGTTATTTCTAAGGATTT

CACCGAAGATAGAATCTGGTTGAACGGTAGAGAAGAAGATGTTGGTCAACCTAGATTGCAAGCCTGTTTGAGAGAAATT

AGATGCTTGGCTAGAAAGAGAAGAAACTCCAGAGATGGTGATCCATTGCCATCTTCATTGTCTTGTAAAGTTCATGTTG

CCTCCGTCAACAATTTTCCAACTGCTGCTGGTTTGGCTTCTTCAGCAGCTGGTTATGCTTGTTTAGCTTATACTTTGGC

TAGAGTCTACGGTGTTGAATCCGATTTGTCTGAAGTTGCTAGAAGAGGTTCTGGTTCAGCTTGTAGATCATTATACGGT

GGTTTTGTCGAATGGCAAATGGGTGAACAAGCTGATGGTAAAGATTCCATTGCTAGACAAGTTGCTCCAGAATCTCATT

GGCCAGAATTGAGAGTTTTGATTTTGGTTGTTTCCGCCGAAAAGAAGTTGACTGGTTCTACTGTTGGTATGAGAGCTTC

TGTTGAAACATCTCCTTTGTTGAGATTCAGAGCCGAATCTGTTGTTCCAGCTAGAATGGCTGAAATGGCTAGATGTATT

AGAGAAAGAGACTTCCCATCTTTCGCTCAATTGACTATGAAGGACTCCAATCAATTCCATGCTACTTGTTTGGATACCT

TCCCACCAATTTCTTACTTGAACGCTATTTCTTGGAGAATCATCCATTTGGTCCATAGATTCAATGCTCATCATGGTGA

TACCAAGGTTGCTTACACTTTTGATGCTGGTCCAAACGCTGTTATTTTCACTTTGGATGATACCGTTGCTGAATTTGTT

GCAGCTGTTTGGCATGGTTTTCCACCAGGTTCTAACGGTGATACTTTTTTGAAGGGTTTACAAGTTAGACCAGCTCCAT

TGTCTGCTGAATTGCAAGCTGCTTTAGCTATGGAACCTACTCCAGGTGGTGTTAAGTATATTATCGTTACTCAAGTTGG

TCCAGGTCCACAAATTTTAGATGATCCATGTGCTCATTTGTTGGGTCCAGATGGTTTGCCAAAACCTGCTGCTTAATGA

CTGCAG

## Synthetic *BmMVD*

GGATCCATGTCTAGTAACGATGGTTCTTCTGAT

AGAGTCAGAGAAGTTAAGGTTATTGCCCCAATCAATATCGCCTTGGTTAAGTATTGGGGTAAGAGAAACGAAGATTTGA

TGTTGCCATTGAACGATTCCATTTCTTTGTCCATCAACGATATGTGTGCTAAGACCAGAGTTAGAATTGGTGCTTCTGT

TAAGAAGGACTCCGTTTCTATTAACGGTTCTAACGTCTGCTTGTCTAAACATCCAGGTTTCTTGAGATGCTTCAAAGAA

GTCAGAAGATTGATCAGAAAGAGATCCATCATTTCTGAAACCGCTGGTAAATCCGAAAAGCACGATTACTTTTCCAAGT

TCGAAGTCGTCAGTGAAACTAACTTTCCAATTGAAGCTGGTTTGGCTTCTTCTGCTGCTGGTTTTGCTGCTATTGCTTA

TGGTTTGGGTCAAATCTACCAATTGAACATCTCCGATATTATCAGAGTTGCCAGAATGGGTAAACATGCTGTTGCTATC

GTTATCTTGAAGTGCTCTCAAAACGAATTGAGATTGGACAACTATTCCGGTTCTGGTTCTGCTTGTAGATCTATTTTGT

CTGGTTTGGTTCATTGGAAAGCTGGTACTGCTGAAGATGGTACTGATTGCATTTGTGAAACTGTTTTCCCAGAAGATTA

CTGGCCAACTTTGAGATCCTTGATTTTGGTTACTTCCCACGGTACTAAGAAAGTCTCTTCTTCTAATGGTATGCAATCC

ACCGTTAAGACCTCTAAGTTGTTGCAAGCTAGAATGGATATTGTCCCAGAACAAATCACCAAGTTGAGAAACGCTTTCA

GAGACAGAAACTTCGAACAATTGGCCAAGGTTATCATGTCTGATTCTGGTCAATTGCATGCTTTGTGTATGGATACAAT

GCCATCCTTGAGATACTTGAACGATAACTCTTGGTACTTGATGCAATTGATCCATGCTTTGAACAGACATTGCAAGGAT

ACAAAGGTTGCTTACACTTTTGATGCTGGTCCAAACTGTTGTTTGTTCTTGGAATCTGTTAACGTCCCATTGATATTGG

CTGCTGTTAACAAATACTGCAAATTGAGATCCGACTTGATTGAAAGAGTTGCTAAATACCCAGCTGCTTTCGAATACGG

TAATTTGAGATCTTTGGTTGAAGAAGAACAAAAGAACTTGGTCTTGTTCGAATCCATTGACGGTCAAGAAAACTCCGAA

ATTGAACCATTGGATGGTGTTGTTAACGACATCTTCTTTTCTTGTGTTGGTGTTGGTCCATTCTTGGCTGAATCTAGAT

AATGACTGCAG

## Synthetic *HsKRS1*

GGATCCATGTTGACTCAAGCTGCTGTTAGATTG

GTTAGAGGTTCTTTGAGAAAAACCTCTTGGGCTGAATGGGGTCATAGAGAATTGAGATTGGGTCAATTGGCTCCTTTTA

CTGCTCCACATAAGGATAAGTCTTTCTCAGACCAAAGATCTGAATTGAAGAGAAGATTGAAGGCCGAAAAGAAGGTTGC

TGAAAAAGAAGCCAAGCAAAAAGAATTGTCCGAAAAGCAATTGTCTCAAGCTACTGCTGCTGCTACTAATCATACAACT

GATAATGGTGTTGGTCCAGAAGAAGAATCCGTTGACCCAAATCAATATTACAAGATCAGATCCCAAGCCATCCACCAAT

TGAAAGTTAATGGTGAAGATCCATACCCACACAAGTTCCACGTTGATATTTCTTTGACCGACTTCATCCAAAAGTACTC

TCACTTGCAACCAGGTGATCATTTGACTGATATTACCTTGAAAGTTGCCGGTAGAATCCATGCTAAAAGAGCTTCAGGT

GGTAAGTTGATCTTCTACGATTTGAGAGGTGAAGGTGTTAAGTTGCAAGTTATGGCTAACTCCAGAAACTACAAGTCTG

AAGAAGAATTTATCCACATCAACAACAAGTTGAGAAGAGGTGATATCATCGGTGTACAAGGTAATCCAGGTAAAACTAA

GAAGGGTGAATTGTCCATTATCCCATACGAAATCACTTTGTTGTCTCCATGCTTGCATATGTTGCCACACTTGCATTTT

GGTTTGAAGGACAAAGAAACCAGATACAGACAAAGATACTTGGACTTGATCTTGAACGACTTCGTCAGACAAAAGTTCA

TCATCAGATCCAAGATCATCACCTACATTAGATCTTTCTTGGACGAATTGGGTTTCTTGGAAATTGAAACCCCAATGAT

GAACATCATTCCAGGTGGTGCTGTTGCTAAACCATTCATTACTTACCACAACGAATTGGACATGAACTTGTACATGAGA

ATCGCCCCAGAATTATACCACAAGATGTTGGTTGTTGGTGGTATCGATAGAGTTTACGAAATCGGTAGACAATTCAGAA

ACGAAGGTATCGATTTGACCCACAACCCAGAATTTACTACCTGCGAATTTTACATGGCTTACGCCGATTATCACGACTT

GATGGAAATTACCGAAAAGATGGTATCCGGTATGGTTAAGCACATTACTGGTTCTTACAAGGTTACCTATCATCCAGAT

GGTCCTGAAGGTCAAGCCTATGATGTTGATTTTACTCCACCTTTTAGAAGAATCAACATGGTTGAAGAATTGGAAAAGG

CCTTGGGTATGAAGTTGCCAGAAACAAACTTGTTCGAAACCGAAGAAACTAGAAAGATTTTGGACGATATTTGCGTTGC

TAAGGCTGTTGAATGTCCACCACCAAGAACTACTGCTAGATTATTGGATAAGTTGGTTGGTGAATTTTTGGAAGTTACC

TGCATTAACCCAACCTTCATTTGTGATCATCCACAAATCATGTCTCCATTGGCTAAATGGCACAGATCTAAAGAAGGTT

TGACCGAAAGATTCGAATTATTCGTCATGAAGAAAGAAATCTGCAACGCCTACACCGAATTGAATGATCCAATGAGACA

AAGACAATTATTCGAAGAACAAGCTAAAGCCAAAGCTGCTGGTGATGACGAAGCTATGTTTATTGACGAAAACTTCTGT

ACCGCTTTGGAATATGGTTTGCCACCAACTGCTGGTTGGGGTATGGGTATTGACAGAGTTGCTATGTTCTTGACCGATT

CCAACAACATCAAAGAAGTCTTGTTGTTCCCAGCTATGAAGCCTGAAGATAAGAAAGAAAACGTTGCTACTACCGACAC

TTTGGAATCTACTACTGTTGGTACTTCCGTCTGATGACTGCAG

## Synthetic *BmKRS1*

GGATCCATGATTGCCAACATCGTTAGAAGAGTT

CAATTGGATTGTCACGCCGTTTCTAGATCTTTGTTGAGATACGTTAACGGTTCCTCTGTTACCCAAAAGAGATGTGAAT

CTGAACAAAAGAGACAATTGAAGGCCCAACAAAAGTTGAAAGAAAAGGCCGAAAAAGAATTGCAAAGAGCTGCTGCTGC

TACAGCTGCTACTACTATGGAAAAAGGTAAGAGAGATGAAAAGATGGTTGATCCATCTGACCCACAAGAATACTTCAAG

TTGAGAACCACCTTGATCAACGAAAGAAGATCCAAAGGTATTAACCCATACCCACATAAGTTCCACGTTTCCATTTCTT

TGACCGACTTCATCGAAAAGTACGATTCCTTGGAAAAGGACGTTATCTTGAACGACTCCATCCAAAGAAGAATCTTCTC

CAAGAGAGAATCTGGTGGTAAGTTGATTTTCTACGACTTGCATGGTGAAGGTACTAGATTGCAAGTTTTGGCTAATGCC

AGATTCCATTCTGGTGATGAATCTTTCGATTCATTGCACGACAGAATCAAGAGAGGTGATATTATTGGTGTCAATGGTT

ACCCAGCTAGATCTAAATCTGGTGAATTGTCCATTATCCCAAGAGAAATCGTTCAATTGACTCCATGCTTGCATATGTT

GCCACATACTCATTTCGGTTTGAAAAATCAAGAAACCAGATACAGAATGAGATACTTGGACTTGATCATGAACACCGAT

GTCAAGAACAAGTTCGTTACTAGATCCAGAACCATTTCATTCTTGAGAAGATATTTGGACAACTTGGGTTTCTTGGAAG

TTGAAACTCCAATGATGAACTTGATTGCTGGTGGTGCTACTGCTAAACCATTCATTACTCATCACAACGATTTGGACAT

GGACTTGTATTTGAGAGTTGCCCCAGAATTATACTTGAAGATGTTGGTTGTTGGTGGTATCGATAGAGTTTACGAAATC

GGTAGAGTTTTCAGAAACGAAGGTATCGATCAAACCCACAATCCAGAATTTACCACCTGTGAATTTTACATGGCTTACG

CCGATTACGAAGATTTGATGAAGATTACCGAAGATATGTTGTCCAGATTGGTCTACTCTATCCATGGTACTTACAAGAT

CCAATACCATCCAAATGGTATCGGTGAAGAACCTGTTTACGAAGTTGACTTTACTCCACCATTCCAAAGAGTTGATATC

TACGACGGTTTACAAGAAAAGTTGGGTGTTAAGTTTCCACCAGCTAACACTTTGGATACTGATGAAGCTAACAAGTTCT

TCGATAAGTTGGCCGTTGAAAACAACGTTGAATGTCCAGCTCCAAGAACTACTGCTAGATTATTGGACAAGTTGATCGG

TGAATTTTTGGAACCTACCTTCATCTCACCAACTTTCTTGACTGGTCATCCACAATTGATGTCTCCATTGGCTAAATGG

CATAGATCAATGCCTGGTTTGACTGAAAGATTCGAATTATTCGTCGTCACCAAAGAAATTGTCAACGCTTACACCGAAT

TGAATGACCCATTAACCCAAAGATTGAGATTCGAAGAACAAGCCGAACAAAAACAAGCCGGTGATGATGAAGCACAAAT

TATCGACGAAAACTTCTGTACCGCTTTGGAATATGGTTTGCCACCAACTGCTGGTTGGGGTATTGGTATTGATAGATTG

ACTATGATCTTGACCGATTCCAACAACATCAAAGAAGTTTTGTTCTTCCCAGCTATGAGACCAGACGAAAAGACTACTA

ATTTGTCTAACGAAGGTGGTAACGCCTGATGACTGCAG

## Synthetic *HsCDC21*

GGATCCATGCCAGTTGCTGGTTCTGAA

TTGCCAAGAAGACCATTGCCACCAGCTGCTCAAGAAAGAGATGCTGAACCTAGACCACCACATGGTGAATTGCAATATT

TGGGTCAAATCCAACACATCTTGAGATGCGGTGTTAGAAAGGATGATAGAACTGGTACTGGTACTTTGTCTGTTTTTGG

TATGCAAGCCAGATACTCCTTGAGAGATGAATTTCCTTTGTTGACCACCAAGAGGGTTTTTTGGAAAGGTGTTTTGGAA

GAATTATTGTGGTTCATCAAGGGTTCCACCAACGCTAAAGAATTGTCATCTAAGGGTGTTAAGATTTGGGATGCTAACG

GTTCTAGAGATTTCTTGGATTCTTTGGGTTTCTCCACTAGAGAAGAAGGTGATTTGGGTCCAGTTTATGGTTTTCAATG

GAGACATTTTGGTGCCGAGTACAGAGATATGGAATCTGATTATTCTGGTCAAGGTGTCGACCAATTGCAAAGAGTTATT

GATACCATTAAGACCAACCCAGACGATAGAAGAATTATCATGTGTGCTTGGAACCCAAGAGATTTGCCATTGATGGCTT

TGCCACCATGTCATGCTTTGTGTCAATTTTACGTTGTCAACTCCGAATTGTCCTGCCAATTATATCAAAGATCTGGTGA

TATGGGTTTGGGTGTCCCATTCAATATTGCTTCATATGCTTTGTTGACCTACATGATTGCTCATATCACTGGTTTGAAG

CCAGGTGATTTCATTCATACTTTGGGTGATGCTCACATCTACTTGAACCATATTGAACCATTGAAGATCCAATTACAAA

GAGAACCTAGACCTTTCCCAAAGTTGAGAATCTTGAGAAAGGTTGAAAAGATCGATGACTTCAAGGCCGAAGATTTCCA

AATTGAAGGTTACAATCCACACCCAACCATCAAAATGGAAATGGCTGTTTGATAACTGCAG

## Synthetic *BmCDC21*

GGATCCATGAAGTCATCTGGTGCTCAT

GGTAATGTTATGGGTGATGCTGGTGTTTTGAAGAACGAAGATGAATCTAAGTACTTGGACCAAGTCAGATATATCTTGA

AGAACGGTGAAAGAATCGATGATAGAACTGGTGTTGGTACTATCTCTGTTTTCGGTATGCATTCCGTTTACTCATTGAG

AAATGGTGTTGTTCCAGTTTTGACCACCAAAAGGGTTTACTGGAAAGGTGTCGTCGAAGAATTATTGTGGTTTATCAGA

GGTGATACCAACGCTAAACACTTGTCTGAAAAGGGTGTTAGAATTTGGGATGCTAACGGTTCTAGACAATTCTTGGATC

AATGTGGTTTCTCCGATAGATCTGAAGGTGATTTGGGTCCAATCTATGGTTTTCAATGGAGACATTGTGGTGCTGAGTA

TAGAGGTATGGATACCGATTACACCAATCAAGGTATCGACCAATTGTCCGAAATCATCGATTTGATCAAGAACGAACCA

CACTCCAGAAGAATTATCTTGTCTGCTTGGAACGTTAAGGACTTGAAATTGATGGCTTTGCCACCATGTCATACTTTGG

CTCAATTTGCTGTTAGAAACGGTGAATTGTCCTGCCAATTATACCAAAGATCTGGTGATATGGGTTTGGGTGTTCCATT

CAATTTGGCTTCATATGGTTTGTTGACCCATATGATTGCTCATGTCTGTGGTTTGAAAACCGGTCATTTGTGTCATGTT

TTAGGTGATGCTCACGTTTACATGAATCATGTTGATGCCTTGCAAGAACAATTGAAGAGACAACCTAGACAATTCCCAA

CCGTTAGATTCATTGGTAACATCAAGACCATTGACGACTTCACCTATGAATCCATCGTTTTGGAAAACTACCAACCTAT

GCCAGCTATTAAGATGGCTATGGCTGTTTGATGACTGCAG

## Synthetic *BmDYS1*

GGATCCATGGACAACGGTAACTGTAAA

TTCGATGTTCATATCGCCGAAATGTCCGTCTTGAAGAAATCTTCTACTATGCCAGCTGATTCCACCATTATTAAGGGTT

ACGATTTCAACGAAGGTATCAACTACGATGCCTTGTTGGACCAATATATGTCTACTGGTTTTCAAGCCTCTCATTTCGC

TCAAGCTGTTCAACAAATCAACACCATGTTGACCATCAGAGAAGAACAATTCGAAGGTGATCATACTTTGCCATACCCA

GAAGGTAAACAAAAAAGAGCTTGCACCATTTTCTTGGGTTACACCTCTAATTTGGTTACCTCTGGTGTTAGAGAAAACA

TCAGATACTTGGTTGAACACGATTTGGTTGATTGCATCGTTACATCTGCTGGTGGTGTTGAAGAAGATTTGATTAAGTG

TTTGGCCCCATCTTACTTGGGTGCTTTTGATTTGGATGGTAAGACCTTGAGACATAACGGTTTGAATAGAGCCGGTAAC

ATTATCATCCCAAACAACAACTACTGCCAATTTGAAGATTGGTTGATGCCAATCTTGGACTCTTGTGAATTGGAACAAA

AGAACAACGATTTCTCTTGGACCCCATCCAAGTTGATTGATAGATTGGGTGCTGAAATCAACGACAAGAGATCTATTTG

TTACTGGGCCCATAGAAACAGAATCCCAGTTTTTTCACCAGCTTTGACCGATGGTTCTATTGGTGATATGTTGTACTTC

CACTCTTTCAGAAACGGTGGTATCAAGTTGGATATCGTCGAAGATTTGAGACACATTAACACTATGGCTGTCAGATCTA

ACAGAACCGGTGTTATTTTGTTGGGTGGTGGTGTAATGAAGCACCATATTAACAATGCTAACTTGATGAGAAACGGTTC

CGATTACGCTGTTTACGTTAATACCGGTCAAGAATTTGATGGTTCTGATTCTGGTGCTAGACCTGATGAAGCTGTTTCT

TGGGGTAAAGTTAGATCAGATTGCAGACCAGTTAAGATCTATGCTGATGCTACTTTGGTTTTCCCTTTGTTGGTTGCTA

AGACTTTCGCTAGACATGTCCAACAAAAACACTCCGAATTGCAAGAAGCCTGATAACTGCAG

## Synthetic *HsRKI1*

GGATCCATGCAAAGACCAGGTCCATTT

TCTACATTATACGGTAGAGTTTTGGCTCCATTGCCAGGTAGAGCTGGTGGTGCTGCTTCAGGTGGTGGTGGTAATTCTT

GGGATTTGCCAGGTTCTCATGTTAGATTACCTGGTAGAGCACAATCTGGTACTAGAGGTGGTGCAGGTAATACTTCTAC

TTCTTGTGGTGATTCCAACTCTATTTGTCCAGCTCCATCTACTATGTCTAAAGCTGAAGAAGCTAAAAAGTTGGCCGGT

AGAGCAGCTGTTGAAAACCATGTTAGAAACAATCAAGTCTTGGGTATCGGTTCTGGTTCTACTATAGTTCATGCCGTTC

AAAGAATTGCCGAAAGAGTTAAGCAAGAAAACTTGAACTTGGTCTGCATTCCAACATCTTTCCAAGCTAGACAATTGAT

CTTGCAATACGGTTTGACCTTGTCCGATTTGGATAGACATCCAGAAATTGATTTGGCTATTGATGGTGCCGATGAAGTT

GATGCTGATTTGAATTTGATTAAGGGCGGTGGTGGTTGTTTGACTCAAGAAAAAATCGTTGCTGGTTACGCCTCTAGAT

TCATCGTTATTGCTGATTTCAGAAAGGACTCCAAGAACTTGGGTGATCAATGGCATAAGGGTATTCCAATTGAAGTTAT

TCCAATGGCCTACGTTCCAGTTTCTAGAGCTGTTTCTCAAAAGTTTGGTGGTGTTGTCGAATTGAGAATGGCTGTTAAC

AAAGCTGGTCCAGTTGTTACTGATAACGGTAACTTTATCTTGGACTGGAAGTTCGATAGAGTTCACAAATGGTCTGAAG

TTAACACCGCCATTAAGATGATTCCAGGTGTTGTTGATACTGGTTTGTTCATTAACATGGCTGAAAGAGTCTACTTCGG

TATGCAAGATGGTTCCGTTAATATGAGAGAAAAGCCATTCTGCTAATGACTGCAG

## Synthetic *BmRKI1*

GGATCCATGAAGACTCATTCTACTTTG

GATTTGGCTAAAAGAGCTGCTGCTTTTGCTGCTGGTGAACAACATGTTAAGTCTGGTTGTAGAATTGGTGTTGGTTCTG

GTACTACTGCTAAGTTCTTGGTTGAATTTTTGGCCGAAAAGGTTAACGATGGTACTGTTAAGGATATTATCTGCGTCCC

ATCTTCATTCTCTACTAGACAATGGTTGATCGACTACGGTTTACAAGTTATCGACTTGGAAAAGATCTTGGACTTGGAT

TTGTGTATTGATGGTGCTGATGAAGTCGACATTAACTTGAACTGTATTAAGGGTTCTGGTGGTGGTTGTTTGACTCAAG

AAAAAATCGTTCAAACCTGCGCCAAGAAGTTCTACATTATTGCTGATGCTTCCAAGCAATCCGAAAAGTTGGGTGATAG

AAATTTCCCAATCCCAATCGAAGTTGTTCCATTTGGTTATGCCCCAGTTTTGAATTGGATCAAGAGACAAGAAGGTGGT

GAAGTCGAATTGAGAACTAACTCCAAAGAAAAATTGGACCCATTCATCACCGACAACAACAACTTTATTTTGGACTGGA

ACTTCCCAAAGAACAAGTACGTTACTACCGAAGATTTGTCTGCCTTGCATACCAGATTGAAATCTTTGCCAGGTGTTGT

TGAAACTGGTTTGTTTATCGGTGTTGCTGAAAAGGCTTATTTCGCTACTGCTGATGGTAATGTCACCGAAAGATTAAGA

CCAGATCCATCCTTGCACTTTTCTTTGAATCAAAAATCCTCCTTGCATTGATAACTGCAG

# 3. Plasmid used in this study

**Fig A**

**Fig B**

**Fig C**

**Fig D**

**Fig E**

**Fig F**

**Fig G**

**Fig H**

**Fig I**

**Fig J**

**Fig K**

**Fig L**

**Fig M**

**Fig N**

**Fig O**

**Fig P**

**Fig Q**

**Fig R**

**Fig S**

**Fig T**

**Fig U**

**Fig V**

**Fig W**

**Fig X**

**Fig Y**

**Fig Z**

**Fig AA**

**Fig AB**

**Fig AC**

**Fig AD**

**Fig AE**

**Fig AF**

# 3. Strains used in this Study

| **Strain name** | **Plasmid** | **Genotype** | **Source** |
| --- | --- | --- | --- |
| BY4743 | - | *MAT****a****/MATα his3∆1/his3∆1 leu2∆0/leu2∆0 met15∆0/MET15 LYS2/lys2∆0 ura3∆0/ura3∆0* | EUROSCARF |
| BY4741 | - | *MAT****a*** *his3∆1 leu2∆0 met15∆0 LYS2 ura3∆0* | EUROSCARF |
| BY4742 | - | *MATα his3∆1 leu2∆0 MET15 lys2∆0 ura3∆0* | EUROSCARF |
| *nmt1∆/NMT1* | - | *nmt1::KanMX/NMT1 MAT****a****/MATα his3∆1/his3∆1 leu2∆0/leu2∆0 met15∆0/MET15 LYS2/lys2∆0 ura3∆0/ura3∆0* | EUROSCARF |
| *pgk1∆/PGK1* | - | *pgk1::KanMX/PGK1 MAT****a****/MATα his3∆1/his3∆1 leu2∆0/leu2∆0 met15∆0/MET15 LYS2/lys2∆0 ura3∆0/ura3∆0* | EUROSCARF |
| *tpi1∆/TPI1* | - | *tpi1::KanMX/TPI1 MAT****a****/MATα his3∆1/his3∆1 leu2∆0/leu2∆0 met15∆0/MET15 LYS2/lys2∆0 ura3∆0/ura3∆0* | EUROSCARF |
| *sah1∆/SAH1* |  | *sah1::KanMX/SAH1 MAT****a****/MATα his3∆1/his3∆1 leu2∆0/leu2∆0 met15∆0/MET15 LYS2/lys2∆0 ura3∆0/ura3∆0* | EUROSCARF |
| *ipp1∆/IPP1* |  | *ipp1::KanMX/IPP1 MAT****a****/MATα his3∆1/his3∆1 leu2∆0/leu2∆0 met15∆0/MET15 LYS2/lys2∆0 ura3∆0/ura3∆0* | EUROSCARF |
| *sec53∆/SEC53* | - | *sec53::KanMX/SEC53 MAT****a****/MATα his3∆1/his3∆1 leu2∆0/leu2∆0 met15∆0/MET15 LYS2/lys2∆0 ura3∆0/ura3∆0* | EUROSCARF |
| *cdc21∆/CDC21* | - | *cdc21::KanMX/CDC21 MAT****a****/MATα his3∆1/his3∆1 leu2∆0/leu2∆0 met15∆0/MET15 LYS2/lys2∆0 ura3∆0/ura3∆0* | EUROSCARF |
| *krs1∆/KRS1* | - | *krs1::KanMX/KRS1 MAT****a****/MATα his3∆1/his3∆1 leu2∆0/leu2∆0 met15∆0/MET15 LYS2/lys2∆0 ura3∆0/ura3∆0* | EUROSCARF |
| *ade13∆/ADE13* |  | *ade13::KanMX/ADE13 MAT****a****/MATα his3∆1/his3∆1 leu2∆0/leu2∆0 met15∆0/MET15 LYS2/lys2∆0 ura3∆0/ura3∆0* | EUROSCARF |
| *mvd1∆/MVD1* |  | *mvd1::KanMX/MVD1 MAT****a****/MATα his3∆1/his3∆1 leu2∆0/leu2∆0 met15∆0/MET15 LYS2/lys2∆0 ura3∆0/ura3∆0* | EUROSCARF |
| *rki1∆/RKI1* |  | *rki1::KanMX/RKI1 MAT****a****/MATα his3∆1/his3∆1 leu2∆0/leu2∆0 met15∆0/MET15 LYS2/lys2∆0 ura3∆0/ura3∆0* | EUROSCARF |
| *cdc8∆/CDC8* |  | *cdc8::KanMX/CDC8 MAT****a****/MATα his3∆1/his3∆1 leu2∆0/leu2∆0 met15∆0/MET15 LYS2/lys2∆0 ura3∆0/ura3∆0* | EUROSCARF |
| *dys1∆/DYS1* |  | *dys1::KanMX/DYS1 MAT****a****/MATα his3∆1/his3∆1 leu2∆0/leu2∆0 met15∆0/MET15 LYS2/lys2∆0 ura3∆0/ura3∆0* | EUROSCARF |
| *phs1∆/PHS1* |  | *phs1::KanMX/PHS1 MAT****a****/MATα his3∆1/his3∆1 leu2∆0/leu2∆0 met15∆0/MET15 LYS2/lys2∆0 ura3∆0/ura3∆0* | EUROSCARF |
| *pis1∆/PIS1* |  | *pis1::KanMX/PIS1 MAT****a****/MATα his3∆1/his3∆1 leu2∆0/leu2∆0 met15∆0/MET15 LYS2/lys2∆0 ura3∆0/ura3∆0* | EUROSCARF |
| *nmt1∆/NMT1_p* | - | *pdr5::HISMX/PDR5 nmt1::KanMX/NMT1 MAT****a****/MATα his3∆1/his3∆1 leu2∆0/leu2∆0 met15∆0/MET15 LYS2/lys2∆0 ura3∆0/ura3∆0* | [9] |
| *pgk1∆/PGK1_p* | - | *pdr5::HISMX/PDR5 pgk1::KanMX/PGK1 MAT****a****/MATα his3∆1/his3∆1 leu2∆0/leu2∆0 met15∆0/MET15 LYS2/lys2∆0 ura3∆0/ura3∆0* | [9] |
| *tpi1∆/TPI1_p* | - | *pdr5::HISMX/PDR5 tpi1::KanMX/TPI1 MAT****a****/MATα his3∆1/his3∆1 leu2∆0/leu2∆0 met15∆0/MET15 LYS2/lys2∆0 ura3∆0/ura3∆0* | This work |
| *sah1∆/SAH1_p* | - | *pdr5::HISMX/PDR5 sah1::KanMX/SAH1 MAT****a****/MATα his3∆1/his3∆1 leu2∆0/leu2∆0 met15∆0/MET15 LYS2/lys2∆0 ura3∆0/ura3∆0* | This work |
| *ipp1∆/IPP1_p* | - | *pdr5::HISMX/PDR5 ipp1::KanMX/IPP1 MAT****a****/MATα his3∆1/his3∆1 leu2∆0/leu2∆0 met15∆0/MET15 LYS2/lys2∆0 ura3∆0/ura3∆0* | This work |
| *sec53∆/SEC53_p* | - | *pdr5::HISMX/PDR5 sec53::KanMX/SEC53 MAT****a****/MATα his3∆1/his3∆1 leu2∆0/leu2∆0 met15∆0/MET15 LYS2/lys2∆0 ura3∆0/ura3∆0* This work | This work |
| *cdc21∆/CDC21_p* | - | *pdr5::HISMX/PDR5 cdc21::KanMX/CDC21 MAT****a****/MATα his3∆1/his3∆1 leu2∆0/leu2∆0 met15∆0/MET15 LYS2/lys2∆0 ura3∆0/ura3∆0* | This work |
| *krs1∆/KRS1_p* | - | *pdr5::HISMX/PDR5 krs1::KanMX/KRS1 MAT****a****/MATα his3∆1/his3∆1 leu2∆0/leu2∆0 met15∆0/MET15 LYS2/lys2∆0 ura3∆0/ura3∆0* | This work |
| *ade13∆/ADE13_p* | - | *pdr5::HISMX/PDR5 ade13::KanMX/ADE13 MAT****a****/MATα his3∆1/his3∆1 leu2∆0/leu2∆0 met15∆0/MET15 LYS2/lys2∆0 ura3∆0/ura3∆0* | This work |
| *mvd1∆/MVD1_p* | - | *pdr5::HISMX/PDR5 mvd1::KanMX/MVD1 MAT****a****/MATα his3∆1/his3∆1 leu2∆0/leu2∆0 met15∆0/MET15 LYS2/lys2∆0 ura3∆0/ura3∆0* | This work |
| *rki1∆/RKI1_p* | - | *pdr5::HISMX/PDR5 rki1::KanMX/RKI1 MAT****a****/MATα his3∆1/his3∆1 leu2∆0/leu2∆0 met15∆0/MET15 LYS2/lys2∆0 ura3∆0/ura3∆0* | This work |
| *cdc8∆/CDC8_p* | - | *pdr5::HISMX/PDR5 cdc8::KanMX/CDC8 MAT****a****/MATα his3∆1/his3∆1 leu2∆0/leu2∆0 met15∆0/MET15 LYS2/lys2∆0 ura3∆0/ura3∆0* | This work |
| *dys1∆/DYS1_p* | - | *pdr5::HISMX/PDR5 dys1::KanMX/DYS1 MAT****a****/MATα his3∆1/his3∆1 leu2∆0/leu2∆0 met15∆0/MET15 LYS2/lys2∆0 ura3∆0/ura3∆0* | This work |
| *phs1∆/PHS1_p* | - | *pdr5::HISMX/PDR5 phs1::KanMX/PHS1 MAT****a****/MATα his3∆1/his3∆1 leu2∆0/leu2∆0 met15∆0/MET15 LYS2/lys2∆0 ura3∆0/ura3∆0* | This work |
| *pis1∆/PIS1_p* | - | *pdr5::HISMX/PDR5 pis1::KanMX/PIS1 MAT****a****/MATα his3∆1/his3∆1 leu2∆0/leu2∆0 met15∆0/MET15 LYS2/lys2∆0 ura3∆0/ura3∆0* | This work |
| yBmNMT_p | pCM*Bm*NMT | *pdr5::HISMX nmt1::KanMX MATα his3∆1 leu2∆0 MET15 lys2∆0 ura3∆0* | This work |
| ySmmNMT_p | pCM*Sm*NMT | *pdr5::HISMX nmt1::KanMX MATα his3∆1 leu2∆0 MET15 lys2∆0 ura3∆0* | [9] |
| yHsNMT_p | pCM*Hs*NMT | *pdr5::HISMX nmt1::KanMX MATα his3∆1 leu2∆0 MET15 lys2∆0 ura3∆0* | [9] |
| yBmPGK_p | pCM*Bm*PGK | *pdr5::HISMX pgk1::KanMX MATα his3∆1 leu2∆0 MET15 lys2∆0 ura3∆0* | This work |
| ySmPGK_p | pCM*Sm*PGK | *pdr5::HISMX pgk1::KanMX MATα his3∆1 leu2∆0 MET15 lys2∆0 ura3∆0* | [9] |
| yHsPGK_p | pCM*Hs*PGK | *pdr5::HISMX pgk1::KanMX MATα his3∆1 leu2∆0 MET15 lys2∆0 ura3∆0* | [9] |
| yBmTPI_p | pCM*Bm*TPI | *pdr5::HISMX tpi1::KanMX MATα his3∆1 leu2∆0 MET15 lys2∆0 ura3∆0* | This work |
| yHsTPI_p | pCM*Hs*TPI | *pdr5::HISMX tpi1::KanMX MATα his3∆1 leu2∆0 MET15 lys2∆0 ura3∆0* | This work |
| yBmSAH_p | pCM*Bm*SAH | *pdr5::HISMX sah1::KanMX MATα his3∆1 leu2∆0 MET15 lys2∆0 ura3∆0* | This work |
| yHsSAHa_p | pCM*Hs*SAHa | *pdr5::HISMX pgk1::KanMX MATα his3∆1 leu2∆0 MET15 lys2∆0 ura3∆0* | This work |
| yHsSAHb_p | pCM*Hs*SAHb | *pdr5::HISMX pgk1::KanMX MATα his3∆1 leu2∆0 MET15 lys2∆0 ura3∆0* | This work |
| yHsIPPa_p | pCM*Hs*IPPa | *pdr5::HISMX ipp1::KanMX MATα his3∆1 leu2∆0 MET15 lys2∆0 ura3∆0* | This work |
| yBmCDC21_p | pCM*Bm*CDC21 | *pdr5::HISMX cdc21::KanMX MATα his3∆1 leu2∆0 MET15 lys2∆0 ura3∆0* | This work |
| yHsCDC21_p | pCM*Hs*CDC21 | *pdr5::HISMX cdc21::KanMX MATα his3∆1 leu2∆0 MET15 lys2∆0 ura3∆0* | This work |
| yHsMVD_p | pCM*Hs*MVD | *pdr5::HISMX mvd1::KanMX MAT****a*** *his3∆1 leu2∆0 met15∆0 LYS2 ura3∆0* | This work |
| yBmRKI_p | pCM*Bm*RKI | *pdr5::HISMX rki1::KanMX MATα his3∆1 leu2∆0 MET15 lys2∆0 ura3∆0* | This work |
| yHsRKI_p | pCM*Hs*RKI | *pdr5::HISMX rki1::KanMX MATα his3∆1 leu2∆0 MET15 lys2∆0 ura3∆0* | This work |
| yHsCDC8_p | pCM*Hs*CDC8 | *pdr5::HISMX cdc8::KanMX MATα his3∆1 leu2∆0 MET15 lys2∆0 ura3∆0* | This work |
| yBmDYS_p | pCM*Bm*DYS | *pdr5::HISMX dys1::KanMX MAT****a*** *his3∆1 leu2∆0 met15∆0 LYS2 ura3∆0* | This work |
| yBmPIS_p | pCM*Bm*PIS | *pdr5::HISMX pis1::KanMX MAT****a*** *his3∆1 leu2∆0 met15∆0 LYS2 ura3∆0* | This work |
| yHsPIS_p | pCM*Hs*PIS | *pdr5::HISMX pis1::KanMX MAT****a*** *his3∆1 leu2∆0 met15∆0 LYS2 ura3∆0* | This work |

**Table A**

# 4. Scoring growth

**Fig AG**
